# How is the value of the exhibition brand in the eyes of the audience? Based on the perspective of green practice

**DOI:** 10.3389/fpsyg.2022.1019508

**Published:** 2022-09-26

**Authors:** Pengshe Jia, Ying Tang, Yunqian Du

**Affiliations:** ^1^Leisure Sports Research Center, University of Sanya, Sanya, China; ^2^School of Business, Lingnan Normal University, Zhanjiang, China; ^3^School of Tourism, Zhuhai College of Science and Technology, Zhuhai, China

**Keywords:** green practice perception, brand loyalty, exhibition audience, trust-commitment, green self-identity

## Abstract

Studies have found that green practices can help organization create unique competitive advantages, such as enhancing the brand value. However, in the existing research, people did not know much about the exhibition audiences’ perceptions of green practices, or its impact on brand loyalty. This study explores the dimension of green practice perceptions of exhibition audiences, uses the trust-commitment theory to verify the relationship between green practice perceptions and exhibition brand loyalty. A total of 665 valid questionnaires were collected in the two exhibitions. The results show that four of the five types of green practices perceived by the audience will significantly affect green trust and then brand loyalty through commitment. Two of the five types of green practice perception can directly affect brand loyalty. And green self-identity will significantly adjust the relationship between green trust and affective commitment. Finally, suggestions are proposed for academic and practical reference.

## Introduction

As people continue to pay attention to environmental issues, the environmental protection features of products or services are increasingly being valued by consumers. The green practices of enterprises are not only conducive to environmental protection, but also bring positive economic and social benefits to enterprises. Studies have found that companies’ green practices can help create unique competitive advantages (Hart, 1995; Porter and Van der Linde, 1995), such as increasing participants’ willingness to pay premiums (Wong et al., 2015) and enhancing brand value (Li et al., 2018). It can be seen that green operation is one of the important sources of corporate competitive advantages (Lee et al., 2010; Hwang and Lee, 2019).

The exhibition industry is a huge industry. In 2018, there were approximately 32,000 exhibitions directly involving 303 million visitors and nearly five million exhibitors across more than 180 countries (UFI and Oxford Economics, 2019). The environmental impacts of the exhibition industry have been mentioned long ago. Many international organizations have put forward practical guidelines for sustainable events for exhibition organizers. In the exhibition field, audiences are a very important group (Rittichainuwat and Mair, 2012), and the number and structure of audiences are also important contents of exhibition audits (UFI, 2020). At the same time, from the perspective of the competitiveness of exhibition companies, the brand equity of companies should also be based on the perceptions of audiences. However, in the existing research, little is known about the perceptions of green practices of stakeholders such as exhibition audiences. Although the positive relationship between green practices in business operations and brands has been verified by scholars (Chen K. L. et al., 2015; Suki, 2016), some scholars have also begun to pay attention to the subsequent impacts of green events. For example, the impacts of green events on participants’ consumption behavior (Horng et al., 2014; Wong et al., 2015), sustainability awareness (Ahmad et al., 2013) and so on have been discussed. However, few scholars integrate audiences’ perceptions of green practices and exhibition brand loyalty into a framework to explore the influence mechanism between the two.

China’s exhibition industry has developed rapidly in recent years and has ranked first in the world in terms of scale (MCC, 2018). However, what is not commensurate with the scale of industrial development is that there are not many studies in the context of China’s exhibition industry. As consumers of different nationalities or cultural groups have significant differences in their perceptions of environmental sustainability and their impacts (Berezan et al., 2013), in the context of China’s exhibition industry, exploring the relationship between practical audience perceptions of green practices and exhibition brand loyalty has certain theoretical value.

In order to bridge the above research gaps, this research aims to achieve the following research goals: (1) Explore audiences’ perceptions of green practices in exhibitions and their impact on exhibition brand loyalty; (2) Based on the commitment-trust theory, verify the green trust commitment framework’s role between audiences’ perceptions of green practices and exhibition brand loyalty; and (3) Explore the moderating effect of audiences’ green self-identity in the green trust-commitment framework.

## Literature review and hypotheses

### Audience perceptions of green practices in exhibitions

Green practices have been valued in many industries. The exhibition industry is an industry that has a significant impact on the environment. For example, the internationally-renowned organization ISO (2012) pointed out: “Events take a heavy toll on resources, society and the environment, often generating significant waste.” For this reason, green practices have attracted the attention of the exhibition industry. Many environmentally sustainable operations guidelines have been established in the industry for exhibition organizers to learn from, such as the “Green Meeting Guide–Roll out the Green Carpet for Your Participants” (UNEP, 2009). ISO has also developed a green event standard, the ISO, 2012 on Event Sustainability Management. In addition, the American Society for Testing and Materials (ASTM) has launched Standards for Green Meetings. The Global Reporting Initiative has launched Event Organizers Sector Supplement (GRI-EOSS). However, these green practice guidelines are mainly aimed at exhibition organizers. Will exhibition audiences agree that relevant operations are green practices? If there is a difference between the perceptions of the audience and the practices of the exhibition organizer, greenwashing may occur, which may negatively affect the exhibition brand (Chen et al., 2019). It can be seen that discussing green practices in exhibitions from the perspective of audiences has important theoretical and practical significance.

However, there is very little research on the green operation of exhibitions from the perspective of audiences. In the field of event management, few scholars have discussed the green practice perceptions of events such as festivals and conferences. For example, the green practice perceptions of festival participants are defined from the following three facets: green food; green environment and activity; and green design and waste management (Wong et al., 2015). The green practice perceptions of conference participants are understood from the following five aspects: sustainability-related initiatives; electricity consumption; waste diversion; facilities’ food service; and enhancing sustainability (Buathong and Lai, 2017). Due to the significant differences in green practices between trade show events and other events (Merrilees and Marles, 2011), it is necessary to understand the perspectives from which exhibition audiences perceive the organizers’ green practices.

### Green trust

Trust refers to the willingness to rely on an exchange partner in whom one has confidence (Moorman et al., 1992). The concept of trust comes from philosophy and is regarded as the core force of social functions. Smith and Barclay (1997) pointed out that generally people understand trust from two aspects, namely cognitive expectation or affective sentiment and risk-taking behavior or willingness. The act of taking risks is implicit in the expectation of trust (Morgan and Hunt, 1994). Many theories regard trust as a very important core element. For example, the transaction cost theory believes that trust can reduce contract costs (Hill, 1990). The norm of reciprocity in social exchange theory also shows that “the first thing to do is to prove that you are trustworthy” (Blau, 1964). In marketing theory, scholars often regard trust as the core of successful sales (Smith and Barclay, 1997).

With people’s emphasis on green marketing, Chen et al. (2020) proposed the concept of green trust: “a willingness to depend on a product, service, or brand based on the belief or expectation resulting from its credibility, benevolence, and ability about its environmental performance.” It can be seen that green trust also refers to the willingness of consumers to rely on products or services, which comes from the perception and judgment of the company’s environmental performance. Consumers’ decisions are often made under asymmetric information. That is why consumers’ trust often comes from their own perceptions (Kardes et al., 2004), especially the perceptions of quality (McKnight et al., 2004). In terms of green trust, the perceptions of environmental friendliness of products or services will have a significant positive impact on green trust (Chen Y. S. et al., 2015). For this reason, this research proposes:

H1: There is a significant positive relationship between green practices and green trust.

### Green brand commitment

Consumers’ commitment to a brand can be regarded as an emotional or psychological attachment (Warrington and Shim, 2000), which reflects the strength of the brand as the best choice in the series. In the study by Tuškej et al. (2013), brand commitment is considered to be composed of two dimensions, namely, affective commitment and social compliance commitment. Affective commitment refers to consumers’ emotional association with a brand due to positive perceptions of the brand. Social compliance commitment refers to a commitment to a certain product or service based on normative beliefs or the recognition of people around them.

According to the Commitment-Trust Theory, there is a circular relationship between trust and commitment–that is, trust leads to commitment, and commitment strengthens trust. Nevertheless, the influence of trust on commitment should be an earlier relationship (Morgan and Hunt, 1994). The research results of Chen (2010) show that green trust will help consumers and product or service providers form a relationship commitment. This kind of relationship commitment based on green trust may come from one’s own concern for environmental protection, or it may come from the emphasis on environmental protection by social codes of conduct. Based on the above analysis, it can be seen that green trust can have an impact on consumers’ brand commitment. For this reason, this research proposes the following hypotheses:

H2: There is a significant positive relationship between green trust and affective commitment;

H3: There is a significant positive relationship between green trust and social compliance commitment.

### Brand loyalty

Brand loyalty can be defined as the actual pattern of consumers’ purchase behavior for a certain brand (Tucker, 1964), or the behavioral intention for a brand (Assael, 1998). In this research, brand loyalty is defined as consumers’ purchase behavior intentions for a certain brand rather than other brands. Although many scholars have conducted research on the relationship between the product or service provider’s green practice perception and the consumer’s loyalty, they have not reached a unified conclusion. For example, Yusof et al. (2017) found that in non-green hotels, there is no significant relationship between green practice perceptions and consumer loyalty. The research of Martínez (2015) on hotels and that of Ibe-Enwo et al. (2019) on green banks show that green practices directly affect consumer loyalty. Considering that under normal circumstances, consumers’ perceptions of the value of products or services will affect their loyalty to product providers (Cronin et al., 1997), this study believes that there is a significant connection between green practices and loyalty.

Many scholars have pointed out that brand trust is an important antecedent of brand loyalty (i.e., Sahin et al., 2011; Chinomona, 2016). This is mainly because when consumers put trust in a certain company, they will form a dependence on the brand, and then form a positive buying tendency (Lau and Lee, 1999). In addition, brand loyalty and brand commitment are two closely related variables. The difference between them is that loyalty focuses more on behavior, that is, the intention to repeat visits or purchases; while commitment is more inclined to attitude (Assael, 1998). Because attitudes have a decisive influence on behavioral intentions, many scholars define brand loyalty as the behavioral consequences of commitment, and even directly propose the commitment-loyalty link (Pritchard et al., 1999; Fullerton, 2003).

For this reason, this research proposes the following hypotheses:

H4: There is a significant positive relationship between green trust and brand loyalty;

H5: There is a significant positive relationship between affective commitment and brand loyalty;

H6: There is a significant positive relationship between social compliance commitment and brand loyalty;

H7: There is a significant positive relationship between green practices and brand loyalty.

### Moderating effect of green self-identity

Shared values have an important influence on trust and relationship commitment (Mathieu and Zajac, 1990). The so-called shared values refer to the beliefs shared by both parties (Morgan and Hunt, 1994), and are also understood as the appropriateness of a certain behavior that is jointly recognized (Heide and John, 1988). If the theory is extended to the scenario of this research–that is, when the audience and the exhibition organizer have the same environmental values, or both believe that it is appropriate or necessary to take a certain behavior (such as green practices)–trust and relationship commitment are created between the audience and the organizer. The identity theory can be used to explain the correct behavior corresponding to individual roles and how to enhance the status of individuals in society (Callero, 1985). The self-identity here refers to the various meanings given to oneself by self, and positions one in social space through the relationships implied by the identity (Gecas and Burke, 1995). Self-identity is reflected in that person’s beliefs, values, and attitudes (Fisbein and Ajzen, 1975). Therefore, this study introduces self-identity as a green consumer, that is, the degree of concern an individual has about green issues (Carfora et al., 2017). Since the implementation of green initiatives by exhibition organizers reflects their concerns about green issues, when the audience has a strong self-identity as green consumers, the shared values of both parties will also be higher. At this time, the relationship between trust and commitment may be stronger. For this reason, this research proposes:

H8: Green consumer identity has a moderating effect on the green trust-affective commitment relationship;

H9: Green consumer identity has a moderating effect on the green trust-social compliance commitment relationship.

**FIGURE 1:**
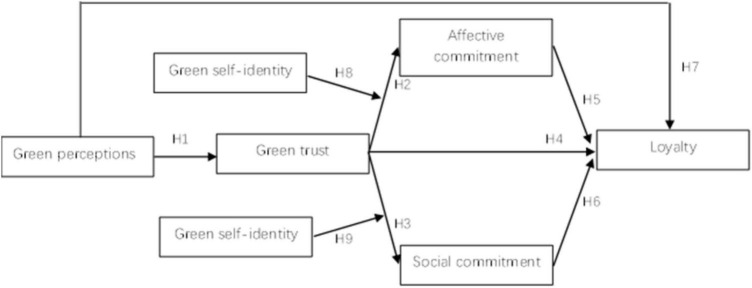
The theoretical framework.

The theoretical framework of this research is shown in Figure 1:

## Materials and methods

### Participants and procedures

This research is divided into two stages. The main purpose of the first stage is to initially construct the dimension of audiences’ perceptions of green practices in exhibitions. In this stage, items were developed using a combination of literature review and in-depth interviews. Researchers conducted a survey of the audience during the 25th Macao International Trade and Investment Fair and conducted a factor analysis of green practice perceptions. In the first phase of the survey, a total of 350 questionnaires were distributed to the audience and 319 valid questionnaires were returned. The main purpose of the second stage is to perform a confirmatory factor analysis of the measurement tools of audiences’ perceptions of green practices, and to verify the theoretical model as well. The survey at this stage was mainly aimed at the audience of the China International Furniture Fair (Guangzhou) in 2021. A total of 400 questionnaires were issued, and 346 valid ones were collected, demographic information is shown in Table 1.

**TABLE 1:** Demographic profile of the sample (*n* = 346).

Demographic	Frequency	Percentage
**Sex**		
Male	208	60.12%
Female	138	39.88%
**Age**		
18–20	11	3.18%
21–30	52	15.03%
31–40	87	25.14%
41–50	105	30.35%
51–60	79	22.83%
≥ 61	12	3.47%
**Education**		
Middle School	9	2.60%
High school	125	36.13%
Undergraduate	178	51.45%
Postgraduate	34	9.82%
**Occupation**		
Employee	165	47.69%
Government staff	56	16.18%
Teaching staff	23	6.65%
Student	49	14.16%
Freelance	32	9.25%
Others	21	6.07%
**Income**		
5,000 ≤	62	17.92%
5,001–7,000	93	26.88%
7,001–9,000	81	23.41%
9,001–11,000	87	25.14%
≥ 11,001	23	6.65%

### Measurements

One of the purposes of this research is to explore audiences’ perceptions of green practices in exhibitions. For this reason, this study constructed the green practice audience perception measurement scale according to the method suggested by Churchill (1979). Based on the compilation of the exhibition industry’s green guide documents and related journal literature, this study interviewed 25 exhibition audience representatives and invited five experts to screen questions. There were a total of 43 items in the initial question bank, of which 12 items were from the industry guidelines, 14 items were from related literature, and 17 items were from in-depth interviews. After two rounds of expert screening, the initial test scale for the first stage was obtained, with a total of 33 items. According to the data analysis results in the first stage, the green practice perception is composed of five dimensions, including a total of 26 items. The five dimensions are: technology based consumption reduction, reduction of exhibition supplies, recycling and reducing emissions, green food service, and strengthening the concept of sustainability. Combined with the data analysis of the second stage, a measurement tool composed of 21 items was finally obtained. For the detailed output of the factor analysis and CFA analysis in the above research process, please refer to the attachment.

Green trust was measured using five items (Chen, 2010). Composite reliability was 0.89. Affective commitment and social compliance commitment were measured using three question items (Tuškej et al., 2013), respectively. Composite reliability was 0.79 and 0.8. Loyalty was measured using three items (Yi et al., 2018). Composite reliability was 0.895. Self-identity as a green consumer was measured using three items (Carfora et al., 2017). Composite reliability was 0.755. All the scale items in the questionnaire were measured with a seven-point Likert scale (where 1 = strongly disagree and 7 = strongly agree).

### Data analysis

This research used SPSS23 for descriptive analysis and factor analysis in the development of measurement tools, and Amos 24 for CFA analysis and SEM analysis. When testing the theoretical model, researchers first checked the kurtosis, skewness, average value and other indicators, and then carried out a common method deviation test to judge whether the data could be further analyzed, after which they analyzed the total correlation among the items of each construct, eliminated inappropriate items, and then checked the reliability. CFA analysis, combination reliability analysis and convergence validity analysis were performed on the measurement model with Amos24.0. On this basis, SEM was used to test the overall structure model and verify the aforementioned hypotheses. This study adopted the bootstrap method to test the mediation effect. The moderated ordinary least squares regression analysis (Aiken et al., 1991) method was adopted to verify the moderating effect.

## Results

Before data analysis, this study checked the missing values and outliers of the data set and found that there were no missing values and outliers. Through kurtosis and skewness indicators, it can be found that the data is normally distributed and suitable for further analysis. Please refer to the attachment for specific data.

### Common method variance

In this study, Harman (1976)’s single factor detection method was used to detect the common method deviation of the data. Through an exploratory factor analysis of all items, if the explanatory variation rate of the unrotated first factor does not exceed 50%, it indicates the common method deviation is within an acceptable range. The explanatory variation of the first factor in this study is 28.73%, which is lower than the 50% standard. It can be seen that there is no covariance bias in the data of this study.

### Measurement model

In this study, SPSS23.0 was used to measure the reliability of each construct in the data set to ensure good consistency in each dimension. Through testing, the Cronbach’s alpha of each construct is between 0.790 and 0.903 (see Table 2), and the overall correlation coefficient (CITC) of the project is all greater than 0.5. Therefore, it can be determined that the data reliability of this study is good. In addition, the results in Table 1 also show that the factor loading of most items is higher than 0.7, the construct reliability value of each dimension is greater than 0.7, and the Average Variance Extracted (AVE) is greater than 0.5.

**TABLE 2:** The results of CFA.

Items		Std.	S.E.	*T*-value	CR	AVE
**TCR**	Cronbach’s α = 0.882				0.883	0.603
TCR6		0.802				
TCR5		0.693	0.058	14.321		
TCR4		0.826	0.058	17.878		
TCR3		0.737	0.058	15.475		
TCR2		0.817	0.053	17.62		
**RES**	Cronbach’s α = 0.885				0.885	0.658
RES5		0.821				
RES4		0.824	0.054	18.223		
RES3		0.825	0.053	18.256		
RES1		0.774	0.057	16.796		
**RRE**	Cronbach’s α = 0.862				0.87	0.692
RRE3		0.829				
RRE2		0.917	0.054	20.906		
RRE1		0.739	0.053	16.165		
**GFB**	Cronbach’s α = 0.851				0.853	0.591
GFB4		0.757				
GFB3		0.768	0.077	15.05		
GFB2		0.807	0.073	15.864		
GFB1		0.743	0.065	14.504		
**SCS**	Cronbach’s α = 0.867				0.871	0.575
SCS5		0.835				
SCS4		0.74	0.052	15.979		
SCS3		0.698	0.059	14.799		
SCS2		0.721	0.058	15.447		
SCS1		0.789	0.055	17.399		
**GrT**	Cronbach’s α = 0.887				0.89	0.62
GT5		0.861				
GT4		0.716	0.062	16.129		
GT3		0.788	0.062	18.614		
GT2		0.746	0.054	17.144		
GT1		0.817	0.056	19.723		
**ABC**	Cronbach’s α = 0.783				0.79	0.56
ABC1		0.625				
ABC2		0.801	0.109	11.71		
ABC3		0.805	0.109	11.736		
**SCBC**	Cronbach’s α = 0.801				0.8	0.572
SCBC3		0.774				
SCBC2		0.802	0.069	13.455		
SCBC1		0.688	0.068	12.27		
**BL**	Cronbach’s α = 0.894				0.895	0.74
BL1		0.836				
BL2		0.839	0.052	19.509		
BL3		0.904	0.053	21.246		
**GS**	Cronbach’s α = 0.754				0.755	0.506
GS3		0.687				
GS2		0.746	0.106	10.199		
GS1		0.701	0.097	10.145		

TCR, technology-based consumption reduction; RES, reduction of exhibition supplies; RRE, recycling and reducing emissions; GFB, green food service; SCS, strengthening the concept of sustainability; GrT, Green Trust; ABC, affective commitment; SCBC, social compliance commitment; BL, Brand Loyalty; Gs, Green self-identity.

The square root of the AVE of each construct in this study is greater than the Pearson correlation coefficient of the construct and the related construct (see Table 3 for details). Therefore, the discriminative validity of each dimension of the scale is generally satisfactory. The model fit of the measurement model also meets the standards of Hair et al. (2010) (see Table 4 for details).

**TABLE 3:** Discriminant validity.

	CR	AVE	TCR	RES	RRE	GFB	SCS	GrT	ABC	SCBC	BL	GS
**TCR**	0.883	0.603	**0.777**									
**RES**	0.885	0.658	0.597	**0.811**								
**RRE**	0.87	0.692	0.629	0.572	**0.832**							
**GFB**	0.853	0.591	0.737	0.714	0.707	**0.769**						
**SCS**	0.871	0.575	0.620	0.581	0.665	0.666	**0.758**					
**GrT**	0.89	0.62	0.593	0.438	0.616	0.624	0.584	**0.787**				
**ABC**	0.79	0.56	0.400	0.359	0.463	0.440	0.495	0.681	**0.748**			
**SCBC**	0.8	0.572	0.314	0.194	0.301	0.262	0.290	0.508	0.499	**0.756**		
**BL**	0.895	0.74	0.479	0.479	0.417	0.555	0.400	0.608	0.518	0.415	**0.86**	
**GS**	0.755	0.506	0.129	0.075	0.039	0.08	0.09	0.152	0.257	0.228	0.231	**0.712**

TCR, technology-based consumption reduction; RES, reduction of exhibition supplies; RRE, recycling and reducing emissions; GFB, green food service; SCS, strengthening the concept of sustainability; GrT, Green Trust; ABC, affective commitment; SCBC, social compliance commitment; BL, Brand Loyalty; Gs, Green self-identity.

**TABLE 4:** The result of model fit.

Index	χ ^2^	Df	χ ^2^/df	RMSEA	SRMR	GFI	AGFI	CFI	NFI	RFI	IFI	TLI
**CFA**	**1027.34**	**620.00**	1.66	0.04	0.04	0.88	0.86	0.95	0.89	0.87	0.95	0.95
**Structural model**	**909.17**	**535.00**	**1.70**	**0.06**	**0.04**	**0.89**	**0.87**	**0.95**	**0.90**	**0.89**	**0.96**	**0.95**
Fitted value	-	-	< 3.0	<°0.05	< 0.05	> 0.9	>°0.9	> 0.9	>°0.9	> 0.9	>°0.9	> 0.9

### Structure equation model

**FIGURE 2:**
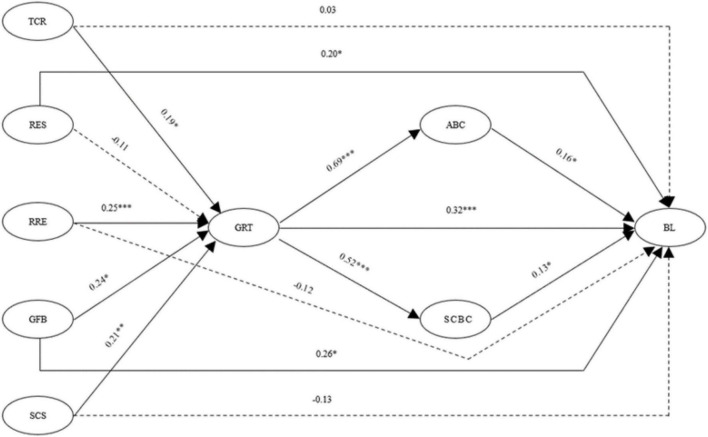
Results of path analysis. TCR, technology-based consumption reduction; RES, reduction of exhibition supplies; RRE, recycling and reducing emissions; GFB, green food service; SCS, strengthening the concept of sustainability; GrT, Green Trust.

This study used the maximum likelihood method to test the hypotheses. Judging from the results of Table 4 (structure model), the model fit is generally satisfactory (Hair et al., 2010). Judging from the results in Figure 2, all hypotheses are supported except that the paths of H1b, H7b, H7c, and H7d are not significant. For detailed data, please refer to Table 5.

**TABLE 5:** The result of structural equal model test.

Hypothesis			Estimate	Std.	S.E.	*T*-value	*P*	Support
RES	→	GrT	−0.147	−0.112	0.093	−1.584	0.113	no
RRE	→	GrT	0.29	0.251	0.086	3.388	***	yes
GFB	→	GrT	0.359	0.243	0.149	2.411	0.016	yes
SCS	→	GrT	0.277	0.209	0.094	2.935	0.003	yes
TCR	→	GrT	0.251	0.193	0.098	2.558	0.011	yes
GrT	→	ABC	0.471	0.693	0.048	9.876	***	yes
GrT	→	SCBC	0.44	0.515	0.05	8.728	***	yes
GrT	→	BL	0.304	0.319	0.091	3.343	***	yes
ABC	→	BL	0.219	0.156	0.102	2.145	0.032	yes
SCBC	→	BL	0.143	0.128	0.064	2.241	0.025	yes
TCR	→	BL	0.043	0.035	0.096	0.448	0.654	no
RES	→	BL	0.249	0.199	0.092	2.708	0.007	yes
RRE	→	BL	−0.137	−0.124	0.085	−1.605	0.108	no
GFB	→	BL	0.359	0.256	0.148	2.423	0.015	yes
SCS	→	BL	−0.158	−0.125	0.094	−1.685	0.092	no

TCR, technology-based consumption reduction; RES, reduction of exhibition supplies; RRE, recycling and reducing emissions; GFB, green food service; SCS, strengthening the concept of sustainability; GrT, Green Trust; ABC, affective commitment; SCBC, social compliance commitment; BL, Brand Loyalty; Gs, Green self-identity. ***P < 0.001.

### Moderation effect

The Hayes process (version 3) was adopted to verify the moderating effect of Green self-identity on the relationship between green trust and affective commitment and social commitment. The data in Table 6 shows that the cross-product term of self-identity as a green consumer and green trust has a significant positive correlation with affective commitment (*t* = 3.413). For this reason, it can be concluded that the relationship between green trust and affective commitment will be moderated by green self-identity, so H8 has been verified. On the other path, the moderating effect of green self-identity is not significant, and H9 is not supported.

**TABLE 6:** Moderation effect of green self-identity.

Dependent variables	Affective commit				Social commit			
	model 1		model 2		model 3		model 4	
	coef.	*t*	coef.	*t*	coef.	*t*	coef.	*t*
Interrupt	1.521	2.400	4.787	12.446	2.639	4.052	4.781	11.913
Control variables								
Gender	0.870	0.768	0.074	0.662	−0.029	−0.249	−0.029	−0.246
Age	−0.041	−0.838	−0.029	−0.599	−0.083	−1.667	−0.083	−1.665
Tourist type	−0.042	−0.704	−0.024	−0.401	0.024	0.399	0.024	0.390
Vocational	−0.030	−1.256	−0.025	−1.041	0.029	1.153	0.029	1.144
Education	0.026	0.482	0.021	0.399	−0.045	−0.828	−0.045	−0.825
Income/per month	−0.028	−0.578	−0.036	−0.765	0.047	0.951	0.047	0.952
Green self-identity (Gs)	0.189	2.259	0.282	3.588	0.083	1.076	0.081	0.983
Independent variable								
Green trust (GrT)	0.444	12.423	0.424	11.872	0.335 9	0.116	0.335	8.994
Moderator								
GrT * Gs			0.158	3.413			−0.003	−0.070
Model statistics								
*R* ^2^	0.333		0.353		0.206		0.206	
R^2^ adj.	0.319		0.333		0.189		0.206	
*F*	23.508		22.781		12.219		10.834	

TCR, technology-based consumption reduction; RES, reduction of exhibition supplies; RRE, recycling and reducing emissions; GFB, green food service; SCS, strengthening the concept of sustainability; GrT, Green Trust; ABC, affective commitment; SCBC, social compliance commitment; BL, Brand Loyalty; Gs, Green self-identity.

**FIGURE 3:**
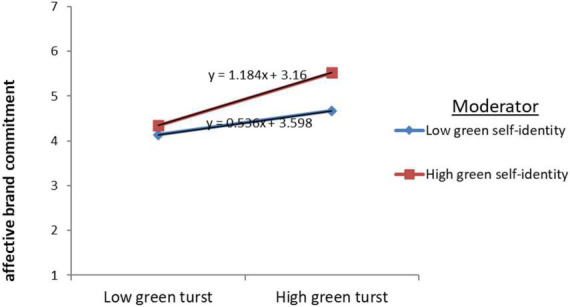
The moderating effect of green self-identity on green trust and affective commitment.

In order to further demonstrate the specific performance of the moderating effect, this studydrew simple slope, as shown in Figure 3. It can be seen that when green self-identity is relatively strong, green trust will lead to a stronger affective commitment.

## Discussion

### Theoretical significance

This study explores the dimension of green practice perceptions in the exhibition industry from the perspective of audiences, uses the trust-commitment theory to verify the relationship between green practice perceptions and exhibition brand loyalty, and draws some enlightening conclusions.

First of all, from the perceptions of exhibition audiences, the green practices in exhibitions can be divided into five dimensions, namely technology-based consumption reduction (TCR); reduction of exhibition supplies (RES); recycling and reducing emissions (RRE), green food service (GFB), and strengthening the concept of sustainability (SCS). This result is consistent with the connotation of the concept of environmental sustainability proposed by Goodland (1995)–that is, it can be understood from two aspects: input and output. Input means to protect resources, while output means to reduce emissions. For example, TCR and RES both represent operations to reduce the use of resources, while RRE and GFB represent the reduction of waste emissions. The content of the SCS dimension in this study is similar to the enhancing sustainability dimension in the study of Buathong and Lai (2017), and GFB is also listed as a dimension in the green practices of events in previous studies (Wong et al., 2015; Buathong and Lai, 2017). It can be seen that audiences’ perceptions of green practices in exhibitions obtained by this research are highly reliable. At the same time, by comparing previous scholars’ research results on green practices in festivals and conferences, it can be seen that participants’ perceptions of green practices in exhibitions are indeed not completely consistent with those in conferences and festivals, which again proves the point of view of Merrilees and Marles (2011).

Second, in terms of the impact of audiences’ perceptions of green practices on green trust, among the five dimensions, four dimensions have a significant impact on green trust, with the exception of RES. This result is not surprising, because in recent years, scholars in the hotel field have also questioned whether green behavior can really guarantee a positive response from consumers (Goh and Balaji, 2016). For example, Chen et al. (2019) pointed out that when hotels ask consumers to make appropriate concessions for environmental reasons, such as reducing the number of towel changes, consumers may question the purpose of the hotels’ environmental behavior, thus forming the impression of greenwashing. The RES in this research refers to the promotion of environmental protection by reducing the supply and consumption of materials. The cost reduction of the organizer and the concession of the audience’s interests are involved. For example, the audience is not provided with paper and pens, and no carpets are used. As a result, the audience doubts the purpose of the exhibition organizer, thus failing to generate green trust. Many scholars have also studied the antecedent variables of trust, and the results show that in order to induce green trust among consumers, it is necessary to improve the quality and performance of environmental protection (Chen and Chang, 2013), cement the relationship between green practices and individuals, strengthen communication (Anderson et al., 1987), etc. It can be seen that SCS has certain advantages in performance presentation and promotion of communication, such as publishing environmental protection regulations and promoting environmental protection booth award. TCR and RRE can enhance the connection between green practices and individuals through audiences’ personal participation, thereby easily generating green trust in audiences.

From the perspective of the relationship between green practice perceptions and exhibition brand loyalty, neither RES, RRE, nor GFB can establish a significant positive relationship with exhibition brand loyalty. The author believes that the uniqueness seeking theory (Ryan, 2008) can explain this finding. Scholars in the fields of fashion, retail, and tourism have verified that consumer loyalty to brands will be affected by brand uniqueness (i.e., Noble et al., 2006; Jun, 2016; Su and Chang, 2018). The content of RRE and GFB practices perceived by the audience in this study, such as the separate recycling program, has become a popular and familiar scene in people’s daily lives (Agovino et al., 2019). For this reason, the lack of unique green practices may lead to the absence of brand loyalty among the audience.

Third, from the perspective of moderating effect of self-identity, green self-identity only has a significant effect on the relationship between green trust and affective commitment but has no significant effect on the relationship between green trust and social compliance commitment. The results show that respondents with higher green self-identity have stronger green trust-affective commitment. This is because when the image of a product is consistent with individuals’ self-concept, it is easier to gain the favor of consumers (Escalas, 2004), thereby forming emotional attachment (Tuškej et al., 2013). From the point of view of its meaning, social compliance commitment is mainly about forming an attachment to a brand based on social norms.

As the exhibition industry has not yet established a unified standard and identification system with a high degree of recognition in the green level of exhibitions, the choice of green brands for exhibition projects is not as obvious as personal environmental behavior in daily life. For this reason, there is no basis for important people around audiences to influence their brand commitment. Therefore, regardless of whether audiences identify themselves with green consumers, the relationship between environmental trust and social compliance commitment is not affected. This finding is also consistent to a certain extent with what Yusof et al. (2017) found in non-green hotels: green practices will not lead to people’s commitment to a hotel brand, because the hotel industry has implemented green certification on a large scale which has been recognized by consumers (Verma and Chandra, 2016). Therefore, for users of non-green hotels, even if consumers perceive a hotel’s green practices, they will not get the support of important people around them to form a brand commitment to the hotel.

### Management significance

The findings of this research also have some inspiration for exhibition management. First of all, as far as exhibition organizers are concerned, actively promoting green practices is not only a manifestation of the organizers’ strong sense of social responsibility, but also helps the audiences to form a certain brand loyalty to the exhibition projects. In particular, the combination of technology and environmental protection and the enhancement of the audiences’ environmental awareness can not only directly increase the brand loyalty to the projects, but also increase the loyalty by enhancing the audiences’ green trust and brand commitment. As for the reduction of pollutant emissions through recycling, exhibition organizers should focus on taking measures to strengthen the link between relevant measures and green trust of audiences. For example, the actual effect of waste recycling can be made explicit, such as notifying audiences of the amount of emission reduction achieved through the recycling process. It is also possible to consider enhancing the association between waste recycling and individuals, such as the association of resource recycling behavior with the personal identities of exhibition participants. In this way, the trust-commitment mechanism can be used to transform the green practices of recycling and emission reduction into brand loyalty in the exhibition industry. In terms of reducing the supply of exhibition materials, organizers should avoid letting audiences form the wrong sense of reducing costs in the name of environmental protection. To this end, organizers can consider establishing multiple communication channels with audiences on relevant green practices to help them build a positive sense of green trust. Secondly, this study also found that the relationship between green trust and social compliance commitment is relatively weak, and that green self-identity does not significantly moderate the above relationship. In view of this, exhibition organizers may also consider actively promoting exhibition projects to pass green certification, so as to let the public understand the environmental protection contributions of the exhibition projects, thereby further enhancing the association between green trust and social compliance commitment, and improving the conversion efficiency from green practices to brand loyalty. Finally, this study found that green self-identity has a significant moderating effect on the relationship between green trust and affective commitment. For this reason, exhibition organizers can also consider enhancing audiences’ green self-identity, so that a series of green practices in exhibitions can enhance audience loyalty to exhibition brands through a stronger trust-commitment framework.

## Research limitations and prospects

Despite some enlightening conclusions, this study also has the following limitations: First, this study’s measurement of audiences’ perceptions of exhibitions’ green practices comes from a self-developed study. Although its reliability and validity have been tested by two exhibition surveys, considering the diversity of exhibition types and content, the universality of exhibition audiences’ perceptions of green practices needs further testing.

Second, in the research process, only a few exhibition projects in Guangzhou and Macau are involved. Due to the cultural differences in people’s perceptions of environmental impacts, the conclusions of related research may also need to be verified in a broader scope.

Third, this research only explores the relationship between the perceptions of green practices and brand loyalty in the exhibition industry from the perspective of audiences. However, there are still many factors that affect audiences’ exhibition brand loyalty, such as the service quality of exhibitions, the service efficiency of exhibitions, etc. Future research can consider integrating these factors and green practices into the model for research.

## Data availability statement

The original contributions presented in this study are included in the article/supplementary material, further inquiries can be directed to the corresponding author.

## Author contributions

All authors listed have made a substantial, direct, and intellectual contribution to the work, and approved it for publication.
